# Effects of ultrasound-guided erector spinae plane block on postoperative acute pain and chronic post-surgical pain in patients underwent video-assisted thoracoscopic lobectomy: a prospective randomized, controlled trial

**DOI:** 10.1186/s12871-023-02100-5

**Published:** 2023-05-09

**Authors:** Jie Zhang, Tong-xin Liu, Wen-xiu Wang, Shu-zhi Zhou, Xin Ran, Peng He

**Affiliations:** 1 Department of Pain Medicine, Zigong Fourth People’s Hospital, Zigong, Sichuan 643000 China; 2 Department of Anesthesiology, North Sichuan Medical College, Nanchong, Sichuan 637000 China; 3 Department of Anesthesiology, Zigong First People Hospital, Zigong, Sichuan 643000 China; 4 Department of Anesthesiology, Ya ’an People Hospital, Ya ’an, Sichuan 625000 China

**Keywords:** Erector spinae plane block, Thoracoscopic lobectomy, Postoperative pain

## Abstract

**Objective:**

To investigate the effects of ultrasound-guided erector spinae plane block (ESPB) on acute and chronic post-surgical pain in patients underwent video-assisted thoracoscopic lobectomy.

**Methods:**

A total of 94 patients, who underwent elective unilateral video-assisted thoracoscopic lobotomy from August 2021 to December 2021 were randomly divided into general anesthesia group (group A, *n* = 46) and ESPB combined with general anesthesia group (group B, *n* = 48) by computer. Patient controlled intravenous analgesia(PCIA) was performed in both groups after operation. The numerical rating scale(NRS) of rest and cough pain at post anesthesia care unit(PACU), 2 h, 6 h, 12 h, 24 and 48 h after operation, frequency of PCIA in 24 h after operation, frequency of rescue analgesia, patient satisfaction, adverse reactions and complications were recorded in the two groups. Incidence of chronic pain at 3 months and 6 months after operation, the effect of daily life and rating of chronic pain management measures were recorded in the two groups.

**Results:**

Compared with group A, rest and cough NRS score at 2 h, 6 h, 12 h, 24 and 48 h after surgery, frequency of PCIA use at 24 h after surgery, frequency of rescue analgesia were significantly decreased in group B (*P* < 0.05). There was no significant difference in NRS scores of rest and cough at PACU after operation between 2 groups after surgery at post anesthesia care unit (*P* > 0.05). There were no significant differences in the incidence of postoperative chronic pain between the 2 groups(*P* > 0.05);The effect of postoperative chronic pain on daily life and pain management measures in group B were significantly lower than those in group A(*P* < 0.05). Compared with group A, patients in group B had higher satisfaction degree, lower incidence of postoperative nausea and vomiting(PONV), and lower incidence of agitation during anesthesia recovery (*P* < 0.05). There were no pneumothorax, hematoma and toxicity of local anesthetic in the 2 groups.

**Conclusion:**

Ultrasound-guided erector spinae plane block can significantly reduce acute post-surgical pain, can not reduce the incidence of chronic post-surgical pain, but can significantly reduce the severity of chronic pain in patients underwent video-assisted thoracoscopic lobectomy.

**Trial registration:**

ChiCTR2100050313,date of registration:26/08/2021

## Introduction

Severe acute pain after thoracic surgery can lead to respiratory complications such as hypoxia, atelectasis and pulmonary infection, which affects the postoperative recovery of patients [1]. Safe and effective postoperative analgesia programs can accelerate enhanced recovery after surgery (ERAS), shorten hospitalization time, reduce hospitalization costs, and improve patient satisfaction [2]. Study had shown that the incidence of chronic post-surgical pain (CPSP) after thoracic surgery is as high as 15–60% [3]. Severe acute pain is an important risk factor for postoperative chronic pain. Good postoperative analgesia method is helpful to reduce the occurrence of chronic post-surgical pain, improve the quality of life of patients after surgery, and has positive significance for patients [4].

Erector spinae plane block is a novel interfascial plane nerve block technique firstly reported by Forero et al. [5] in 2016, which can be used to control postoperative acute pain and treat severe thoracic and back neuropathic pain [6, 7]. This study conducted a randomized controlled trial to investigate the effect of ultrasound guided erector spinae plane block combined with general anesthesia on the postoperative acute and chronic pain in patients undergoing thoracoscopic surgery, so as to provide clinical reference for the selection of a safe and effective postoperative analgesic method.

**Fig. 1:**
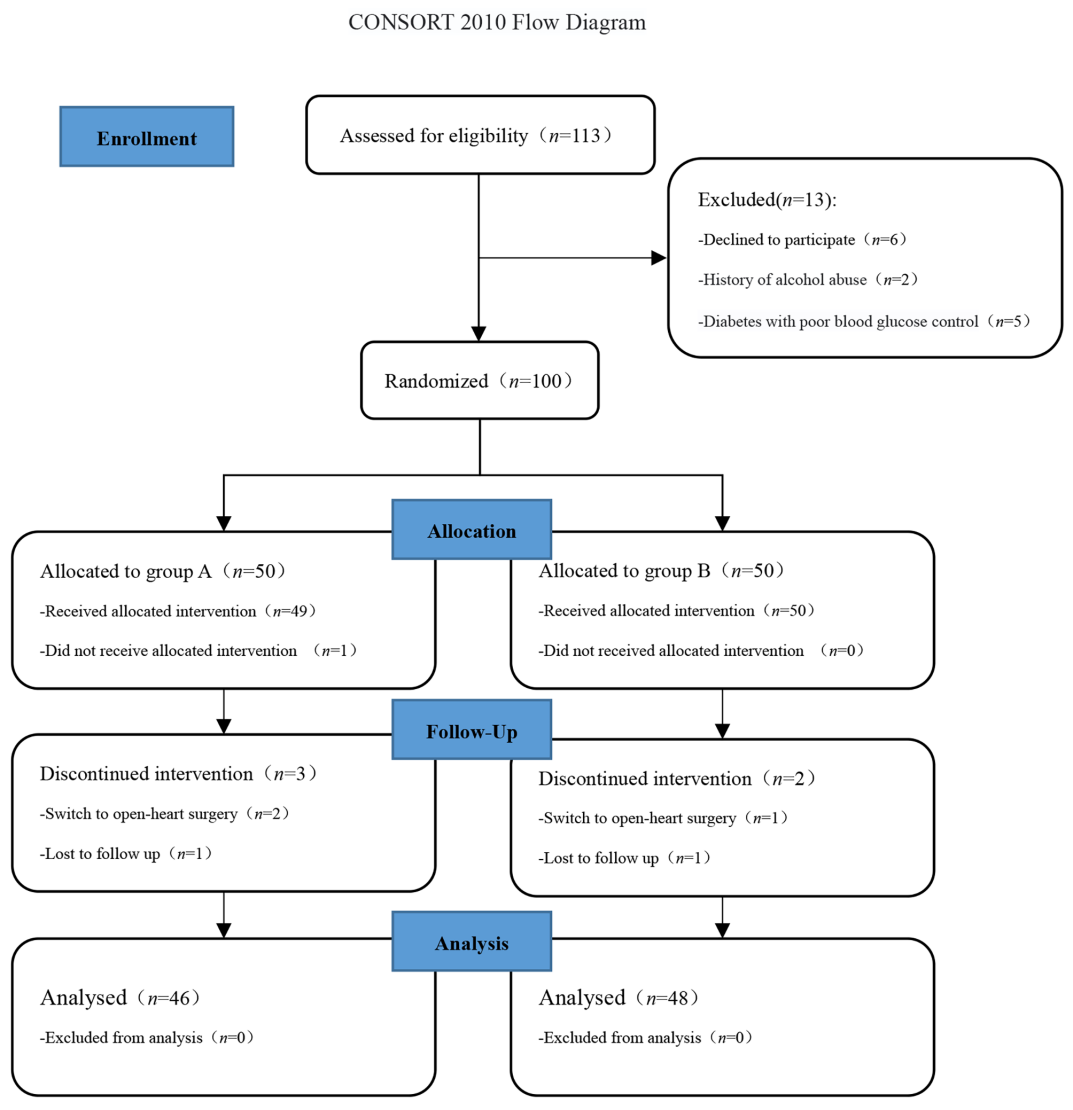
Consolidated Standards of Reporting Trials diagram showing the flow of patients in the study

## Materials and methods

### Research objects

Patients recruitment and flow through the study are described in the Consolidated Standards of Reporting Trials diagram (Fig. 1). This study had passed the ethical review of the Ethics Committee of Ya ‘an People’s Hospital (Identifier: 202111) and completed the Chinese Clinical Trial Registration (The date of registration:26/08/2021,The registration number:ChiCTR2100050313). Patients who underwent elective unilateral thoracoscopic lobectomy in Ya ‘an People’s Hospital from August 2021 to December 2021 were selected for this study. All patients and their family members were informed and signed informed consent. According to the order of admission time, the patients were divided into two groups using random numbers generated by computer (https://www.random.org). The control group was the general anesthesia group (group A), and the experimental group was the ultrasound guided erector spinae plane block combined with general anesthesia group (group B). Inclusion criteria: Patients undergoing elective unilateral thoracoscopic lobectomy; Age 18 to 65 years old; ASA I to III ;Body mass index (BMI) ranged from 18.5 to 28.0 kg/m^2^. Exclusion criteria: Patients and their families refused to participate in the study; History of alcohol and drug abuse, chronic pain, history of neuropathy; Local anesthetics allergy, coagulopathy, injection site infection, diabetes with poor blood glucose control, liver and kidney dysfunction; Peoples with mental illness cannot cooperate with others and have communication difficulties. Withdrawal criteria: patients and their families request withdrawal; Failure of erector spinae plane block under ultrasound guidance; Patients who were converted to thoracotomy; Patients who died during or after surgery; Patients who had serious adverse reactions or who could not follow up records due to accidents.

### Anesthesia technique

The patients and their family members were informed of the anaesthesia related issues and the relevant information of the trial, and signed the informed consent. The patients were trained to use the numerical rating scale (NRS) method to assess pain (0–10 points, 0 is painless, 10 for maximum pain) and postoperative analgesia using a patient-controlled intravenous analgesia pump. Before induction of anesthesia, patients in group B were injected with 0.375% ropivacaine 30 mL on the operation side of T5 by the same experienced anesthesiologist for ultrasond-guided ESPB [7]. Group A did not receive ESPB. Other treatments were the same in both groups. The anesthesia technique was standardized. Radial radial arterial catheter was inserted before induction of general anesthesia to monitor continuous invasive blood pressure, arterial blood gas was obtained through the arterial catheter. Sufentanil 0.3–0.4 µg/kg, propofol 1.5-2.0 mg/kg, and rocuronium 0.8 mg/kg were intravenously induced in the two groups. The position of the double-lumen bronchial catheter was confirmed under bronchoscopy, and the upper edge of the bronchial catheter cuff on the non-ventilated side was confirmed just below the tracheal bulge. Dexamethasone 10 mg and tropisetron 5 mg were intravenously administered for nausea and vomiting prophylaxis before the operation. During the operation, remifentanil 0.1–0.2 µg/(kg·min) and sevoflurane 1-2% were used for anesthesia maintenance. bispectral index (BIS) was maintained at 40–60, and blood pressure and heart rate were maintained at 20% of baseline.Muscle relaxation was achieved by intermittent injections of rocuronium as needed. Single-lung ventilation used a PCV-VG mode with a tidal volume of 5–6 ml/kg, a peak airway pressure of less than 35 cmH_2_O, a respiratory rate of 12–16 per minnte, and PEEP 5cmH_2_O.

All operations were performed by the same thoracic surgeon.The surgical incision was three-holes, with the main operating hole in the anterior axillary line between the 4th and 5th ribs, the thoracoscopic hole in the mid-axillary line between the 6th and 7th ribs, and the auxiliary operating hole in the posterior axillary line between the 8th and 9th ribs. The number of thoracic drainage tubes was one and the location was between the 6th and 7th ribs in the midaxillary line.

PCIA pump was used in both groups after operation, and the drug formulation was: 2 µg/kg sufentanil, 10 mg tropisetron, and normal saline diluted to 150mL. The first dose was 2mL, the background dose was 2mL/h, the PCIA dose was 2mL, the locking time was 10 min, and the maximum dose was 10mL per hour. All patients were connected and started the patient-controlled intravenous analgesia pump 30 min before the end of the operation. In PACU or ward, when patients voluntarily requested analgesia or NRS score ≥ 4, the analgesic pump PCIA was pressed once, and the pain of patients was assessed again 15 min later. If the NRS score ≥ 4, intravenous dezocine 5 mg was given for relief analgesia, and the time of relief analgesia was recorded.

### Outcome measures

The primary outcome was the NRS scores after operation. The NRS scores at rest and cough at 2 h, 6 h, 12 h, 24 and 48 h after operation were observed. Secondary outcomes included the frequency of PCIA use, the frequency of rescue analgesia, the incidence of CPSP, the rating of CPSP effect on daily life, the rating of CPSP management measures, postoperative satisfaction (0–10 points, full score is 10 points), the incidence of agitation during anesthesia recovery, the incidence of PONV, and ESPB-related adverse events.Postoperative follow-up and recording were performed in the ward by the postoperative acute pain management team, which was unaware of the patients’ group. The patients in the two groups were followed up by telephone at 3 months and 6 months after operation. The chronic post-surgical pain (NRS score > 0) was recorded, and the impact of chronic pain on their daily life (no, mild, moderate and severe effects) and the rating of chronic pain management measures (grade A: no treatment measures; Grade B: relief after rest; Grade C: self-purchased medication; Level D: Hospital visits). Telephone follow-up was performed by the same pain management team follow-up member, who was unaware of the patients’ group.

### Statistical analysis

All data were analyzed by SPSS 25.0 software. Measurement data was expressed as mean ± standard deviation ($\bar x$± s), and comparison between groups was performed by t-test. Grade data was expressed as median (1st quartile, 3rd quartile) [M(Q1, Q3)], and comparison between groups was performed by Mann-Whitney U test. Enumeration data was compared by ${\chi ^2}$ test. *P* < 0.05 was considered statistically significant. Statistics were plotted by GraphPad Prism 8 software.

## Results

### Patient demographics and operation characteristics

A total of 100 patients were included in the study, including 1 patient who refused to participate in the study, 5 patients who withdrew midway (3 patients were converted to thoracotomy, and 2 patients were lost during follow-up). The final statistics included 94 patients, 46 in group A and 48 in group B. There were no significant differences in age, height, weight, BMI, gender, ASA classification, operation time and blood loss between the two groups (P > 0.05), as shown in Table 1.

**Table 1:** Patient demographics and operation characteristics

Variables	Group A(*n* = 46)	Group B(*n* = 48)	*t*/${\chi ^2}$-value	*P*-value
Age,years	53.17 ± 5.69	53.31 ± 5.10	0.124	0.901
Height,cm	161.76 ± 6.24	160.63 ± 7.57	-0.792	0.430
Weight,kg	60.67 ± 7.98	60.52 ± 9.03	-0.087	0.931
BMI,kg/m^2^	23.21 ± 2.26	23.38 ± 2.52	0.349	0.728
Male/Female,*n*	25/21	22/26	0.681	0.409
ASA: II/III,*n*	40/6	37/11	1.546	0.214
Duration of surgery,min	120.48 ± 19.97	118.56 ± 22.05	-0.441	0.660
Intraoperative blood loss,mL	161.20 ± 27.55	159.38 ± 32.90	-0.290	0.772

### Postoperative acute pain

**Fig. 2:**
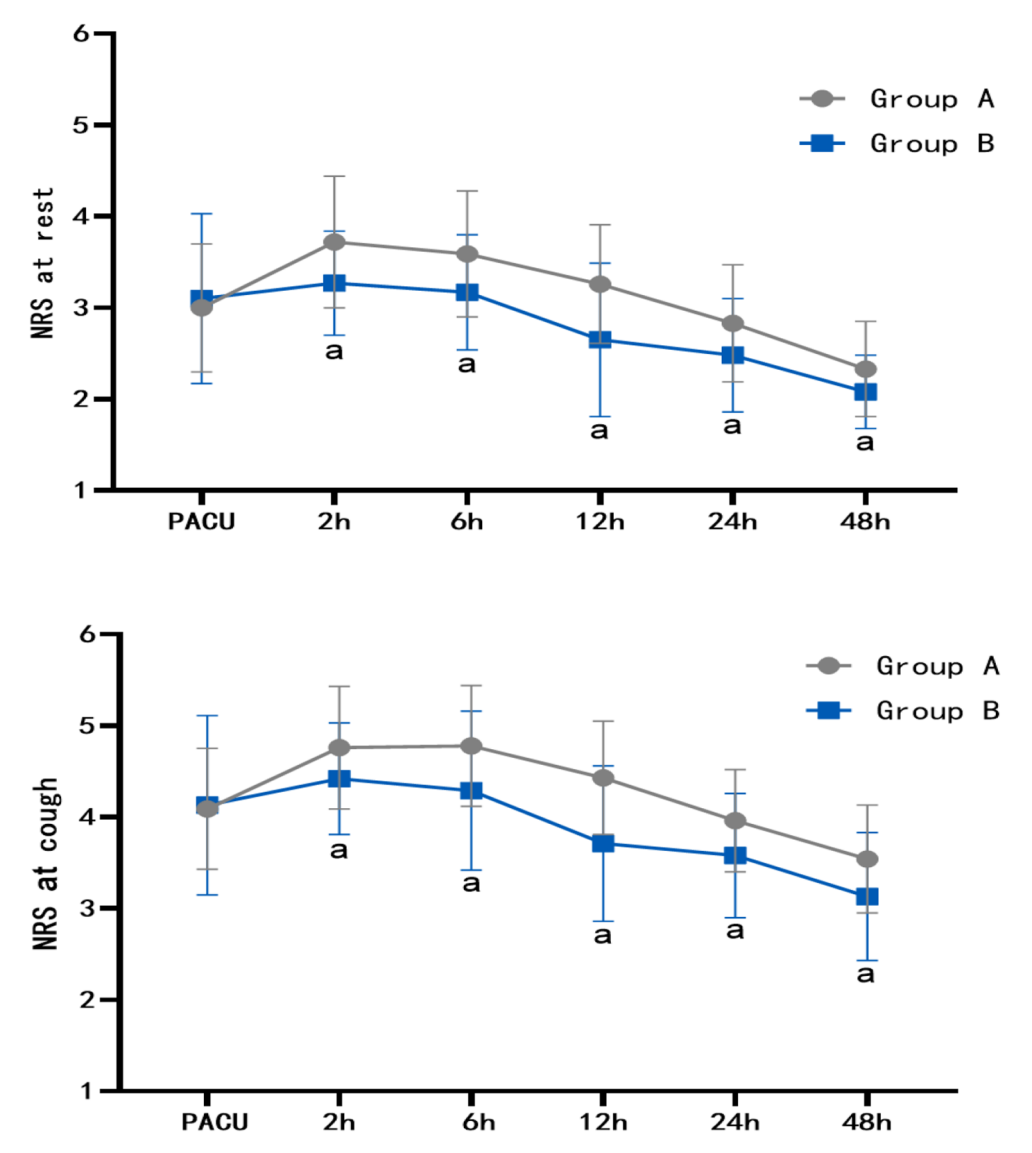
Postoperative numerical rating scale (NRS) pain scores(score, $\bar x$±s) (compared with group A,^a^*P*<0.05)

There was no significant difference at the rest and cough NRS scores between the two groups at the time of PACU admission (P > 0.05). Compared with group A, the rest and cough NRS scores of group B at 2 h, 6 h, 12 h, 24 and 48 h after operation were significantly decreased (P < 0.05), as shown in Table 2; Fig. 2.

**Table 2:** Postoperative numerical rating scale (NRS) pain scores(score, $\bar x$ ±s)

		PACU	2 h	6 h	12 h	24 h	48 h
NRS at rest	Group A(*n* = 46)	3.00 ± 0.70	3.72 ± 0.72	3.59 ± 0.69	3.26 ± 0.65	2.83 ± 0.64	2.33 ± 0.52
	Group B(*n* = 48)	3.10 ± 0.93	3.27 ± 0.57	3.17 ± 0.63	2.65 ± 0.84	2.48 ± 0.62	2.08 ± 0.40
	*t*-value	0.61	-3.33	-3.10	-3.97	-2.67	-2.54
	*P*-value	0.54	0.00	0.00	0.00	0.01	0.01
NRS at cough	Group A(*n* = 46)	4.09 ± 0.66	4.76 ± 0.67	4.78 ± 0.66	4.43 ± 0.62	3.96 ± 0.56	3.54 ± 0.59
	Group B(*n* = 48)	4.13 ± 0.98	4.42 ± 0.61	4.29 ± 0.87	3.71 ± 0.85	3.58 ± 0.68	3.13 ± 0.70
	*t*-value	0.22	-2.59	-3.06	-4.72	-2.91	-3.13
	*P*-value	0.83	0.01	0.00	0.00	0.01	0.00

Compared with group A, The frequency of PCIA use, and the frequency of rescue analgesia at 24 h after operation in group B were significantly lower (P < 0.05), as shown in Table 3.

**Table 3:** PCIA use and rescue analgesia

	frequency of PCIA use,*n*	frequency of rescue analgesia,*n*
Group A(*n* = 46)	4(3, 5)	0(0, 1)
Group B(*n* = 48)	2(1, 3)	0(0, 0)
*t/Z*-value	-4.296	-3.711
*P*-value	<0.001	<0.001

### Chronic post-surgical pain(CPSP)

CPSP occurred in 20 cases (43.5%) in group A and 19 cases (39.6%) in group B at 3 months after operation, and the difference was not statistically significant (P > 0.05). CPSP occurred in 14 cases (30.4%) in group A and 12 cases (25.0%) in group B at 6 months after operation, and the difference was not statistically significant (P > 0.05). The ratings of CPSP effect on daily life and management measures in group B were significantly lower than those in group A at 3 months and 6 months after operation, and the differences were statistically significant (P < 0.05), as shown in Table 4.

**Table 4:** Chronic post-surgical pain

		CPSP,*n(*%)	CPSP effct on daily life				CPSP management measures			
			No	Mild	Moderate	Serious	A	B	C	D
3 months after operation	Group A(*n* = 46)	20(43.5)	3	9	5	3	5	9	4	2
	Group B(*n* = 48)	19(39.6)	10	7	2	0	12	6	1	0
	${\chi ^2}$*/Z*-value	0.147	-2.149				-2.184			
	*P*-value	0.702	0.032				0.029			
6 months after operation	Group A(*n* = 46)	14(30.4)	3	6	4	1	5	5	3	1
	Group B(*n* = 48)	12(25.0)	8	3	1	0	10	1	1	0
	${\chi ^2}$*/Z*-value	0.347	-2.103				-2.305			
	*P*-value	0.556	0.036				0.021			

### Patient satisfaction and adverse reactions

The satisfaction score of group B was higher than that of group A at 24 h after operation, and the difference was statistically significant (P < 0.05). There were 6 cases (12.5%) of agitation during anesthesia recovery in group B, which was lower than 14 cases (30.4%) in group A. There were 4 cases (8.3%) of PONV in group B at 24 h after operation, which was lower than 13 cases (28.3%) in group A, and the differences were statistically significant (P < 0.05), as shown in Table 5. There were no complications of pneumothorax, hematoma and local anesthetic poisoning in the two groups.

**Table 5:** Satisfaction, the incidence of agitation, and the incidence of PONV

	Satisfaction, score	Agitation,*n(*%)	PONV,*n(*%)
Group A(*n* = 46)	8(7, 8)	14(30.4)	13(28.3)
Group B(*n* = 48)	9(8, 9)	6(12.5)	4(8.3)
*Z/*${\chi ^2}$-value	-4.183	4.511	6.296
*P*-value	<0.001	0.034	0.012

## Discussion

In recent years, with the popularization of computed tomography (CT), the detection rate of early lung cancer is increasing [8]. There are many treatment methods for lung cancer patients, and surgical treatment is still one of the main treatment methods. At present, there are thoracotomy and video-assisted thoracic surgery (VATS). With the continuous development of minimally invasive surgical instruments and techniques, VATS has become the main surgical method for early lung cancer. Compared with thoracotomy, VATS has smaller incision, less tissue damage, faster postoperative recovery, higher quality of life and higher satisfaction for patients undergoing VATS [9, 10]. However, due to intercostal nerve injury, intercostal muscle tear, and inflammatory factor release during the operation, moderate to severe pain will still occur after thoracoscopic surgery [11].

ESPB under ultrasound guidance is a new type of interfascial plane block technology, which is considered to be a safer, less invasive and less difficult to operate in the replacement of thoracic epidural block and thoracic paraververtebra block for chest region analgesia [12]. The injection point of ESPB is far away from the pleura, spinal cord and other central nervous regions. A certain volume of local anesthesia is injected between the erector spinae and the transverse process, and the local anesthetic spreads between the fascial planes to block the ventral, dorsal and communicating branches of the corresponding spinal nerves, resulting in analgesic effect [13].

The results of this study showed that compared with patients in group A, patients in group B had lower NRS scores at rest and cough at 2 h, 6 h, 12 h, 24 and 48 h after operation, significantly reduced frequency of of PCIA use and the frequency of rescue analgesia at 24 h after operation, and had higher satisfaction score with pain management. According to the study of Ciftci et al. [14], a single ESPB before surgery can significantly reduce the pain scores at rest and cough at 2 h, 4 h, 8 h, 16 and 24 h after surgery in patients undergoing VATS surgery, and can provide effective analgesia for patients undergoing VATS surgery. The results of this study showed that the NRS scores of patients in group B were still lower at 12 h, 24 h, and 48 h after surgery. The reason may be that the local anesthetic of ESPB spreads through the fascia, and the diffusion is slow and the elimination is slow, which makes the block duration longer. A prospective randomized controlled study of patients undergoing thoracoscopic surgery by Meng Qingsheng et al. [15] found that ESPB could reduce the frequency of of PCIA use and the frequency of rescue analgesia, and had a better analgesic effect. It was found in this study that there was no significant difference at the rest and cough NRS scores between the two groups at the time of PACU admission, which may be due to the fact that the patient-controlled intravenous analgesia pump was connected and opened 30 min before the end of surgery, and the first loading dose of 2mL was given to the patients in this study. The analgesic effect caused by loading dose and the opioids that were not fully metabolized during operation could cover the pain response during PACU, resulting in no significant difference in the NRS scores of rest and cough between the two groups at PACU. These results suggest that ultrasound-guided ESPB has a definite analgesic effect and can effectively control the pain of patients undergoing VATS surgery during and after operation.

The results of this study showed that the incidence of PONV at 24 h after operation and agitation during anesthesia recovery in group B was lower than that in group A, which may be because ultrasound-guided ESPB has a good analgesic effect on patients undergoing VATS surgery, reduces the dosage of opioids, and thus reduces the occurrence of opioid nausea and vomiting side effects. Fields et al. [16] found in their study that patients with agitation during anesthesia recovery were more likely to have postoperative pulmonary complications, which may be related to the functional problems of thoracic drainage tube (disorganized, displaced, blocked, etc.) caused by strenuous exercise during agitation. The study of Shim et al. [17] found that ultrasound-guided ESPB can reduce the incidence of agitation during anesthesia recovery and reduce the incidence of postoperative PONV in patients undergoing VATS surgery. Therefore, it is of great significance for ultrasound-guided ESPB to reduce the incidence of agitation during thoracic surgery anesthesia recovery.

Chronic post-surgical pain (CPSP) refers to the pain that persists for 3 months or more after operation. The pain is mainly confined to the area of surgical injury or the corresponding innervated area, and it is necessary to exclude the pain problems existing before surgery, and the pain caused by infection and tumor recurrence [18]. CPSP is often accompanied by the characteristics of neuropathic pain such as hyperalgesia, dyspnoea, burning sensation or acupuncture sensation, which is a mixed pain including neuropathic pain, and often seriously affects the quality of life of patients [3]. Severe acute pain is an important risk factor for chronic post-surgical pain. Good postoperative acute pain method is helpful to reduce the occurrence of chronic post-surgical pain, improve the quality of life of patients, and has positive significance for patients [19]. This study found that there is no difference between two groups of patients with postoperative incidence of chronic pain, considering the a lot of risk factors for chronic post-surgical pain occurs, mainly includes the six major categories (demographic, genetic susceptibility, complications, pain, and psychological factors of operation), the related risk factors for CPSP between is not independent of each other, but related. A single risk factor cannot determine the occurrence of CPSP [20]. The effect rating of CPSP on daily life and the rating of CPSP treatment measures can indirectly reflect the severity of CPSP in patients [21]. In this study, group B patients postoperative chronic pain rating effect on daily life and treatment of chronic pain rating is significantly lower in group A, the reason may be that regional analgesia techniques such as ESPB combined with PCIA can regulate each site of the pain pathway through different mechanisms to control pain more effectively, reduce the severity of CPSP, and improve the quality of life of patients.It can reduce the severity of CPSP and improve the quality of life of patients [4]. The study of Shi Rong et al. [22] showed that serratus anterior plane block combined with PCIA could reduce the postoperative acute pain of patients with VATS, but could not reduce the incidence of CPSP, but could reduce the severity of CPSP. It is suggested that anesthesiologists should pay attention to the multiple risk factors of CPSP. Perioperative multimodal analgesia can effectively reduce the severity of CPSP.

There are some shortcomings in this study. First, this study used a single ultrasound-guided ESPB with a unilateral injection of 0.375% ropivacaine 30mL at the T5 segment. No comparative study was performed on other concentrations of ropivacaine, other doses, other local anesthetics, and continuous ESPB infusion. Second, this study only compared two analgesia methods, ESPB combined with general anesthesia group and general anesthesia group, and did not compare with other analgesia methods for patients undergoing thoracoscopic lobectomy (such as epidural block, thoracic paravertebral block, serratus anterior plane block, etc.), which had certain limitations. Third, We did not analyze the pathological type of the tumor and whether patients underwent chemotherapy or radiotherapy 3 or 6 months after the operation. Therefore, we were unable to explore the effect of differences in chemotherapy or radiotherapy on CPSP between the two groups of patients, and such differences may have distorted the results of this study. Further studies are required to address these limitations.

## Conclusion

Ultrasound-guided erector spinae plane block can significantly reduce acute post-surgical pain, can not reduce the incidence of chronic post-surgical pain, but can significantly reduce the severity of chronic pain in patients underwent video-assisted thoracoscopic lobectomy.

## Data Availability

The datasets used during the current study are available from the corresponding author on reasonable request.

## Abbreviations

ASA: American Society of Anesthesiologists
CPSP: Chronic post-surgical pain (CPSP)
ERAS: Enhanced recovery after surgery
ESPB: Erector spinae plane block
NRS: Numerical rating scale
PACU: Post anesthesia care unit
PCIA: Patient controlled intravenous analgesia
PONV: Postoperative nausea and vomiting
